# Infrared Thermography as a Complementary Diagnostic Tool in Pediatric Acute Epididymo‐Orchitis: Characterization of the Thermal Differential Pattern

**DOI:** 10.1155/aiu/5411884

**Published:** 2026-11-13

**Authors:** José Alfredo León Zamudio, Onésimo Avilés González, Manuel López, Alma Patricia González

**Affiliations:** ^1^ Pediatric Surgery and Pediatric Urology Services Hospital General Centro Médico Nacional La Raza Instituto Mexicano Del Seguro Social (IMSS) Mexico City Mexico imss.gob.mx; ^2^ Department of Pediatric Surgery, Cleveland Clinic, Cleveland, Ohio, USA, clevelandclinic.org; ^3^ Case Western Reserve University School of Medicine, Cleveland, Ohio, USA, case.edu; ^4^ IMSS Unidad Médica, León, Guanajuato, Mexico

**Keywords:** acute scrotum, infrared thermography, pediatric emergency, pediatric epididymo-orchitis, scrotal ultrasound, thermal differential

## Abstract

**Background:**

Acute epididymo‐orchitis (EO) is one of the most frequent urological emergencies in the pediatric population. Scrotal Doppler ultrasonography (US) is the current diagnostic gold standard; however, it carries well‐recognized limitations in early presentations and in resource‐limited settings. Infrared thermography (IRT) has emerged as a noninvasive, radiation‐free complementary tool capable of objectively detecting inflammatory thermal changes.

**Objectives:**

To determine whether a statistically significant thermal differential exists between the affected testis and the contralateral healthy testis in pediatric patients with acute EO, to characterize its evolution according to the number of days of clinical symptoms, and to evaluate its agreement with scrotal Doppler ultrasound findings.

**Methods:**

Prospective, descriptive, longitudinal, and comparative clinical study conducted from January 1, 2024, to January 31, 2026, at the Pediatric Surgery and Pediatric Urology Services of Hospital General “Centro Médico Nacional La Raza,” IMSS, Mexico City. Seventy‐two consecutive pediatric patients (ages 6–17 years) were enrolled. Thermographic temperatures were recorded at the lower and upper poles of both testes. Paired Student’s *t*‐test and Pearson correlation coefficient were used for analysis.

**Results:**

The mean age was 13.58 ± 2.56 years. The left testis was most commonly affected (54.2%). Mean temperature of the affected testis was significantly higher at both the lower pole (33.96°C vs. 32.28°C; *p* < 0.001) and the upper pole (34.42°C vs. 32.28°C; *p* < 0.001). Mean thermal differential (ΔT) was 1.68°C at the lower pole and 2.14°C at the upper pole. A positive correlation was observed between ΔT and days of evolution (*r* = 0.24–0.25; *p* < 0.05). In the 11 patients (15.3%) with nondiagnostic ultrasound, thermography documented a positive thermal differential in all cases.

**Conclusions:**

IRT identifies a statistically significant thermal differential between the affected and healthy testes in pediatric EO, characterizing a consistent thermal pattern associated with the inflammatory process. It is particularly useful as a complementary tool in cases where Doppler ultrasound is inconclusive. This study is best interpreted as a descriptive characterization of the thermal pattern of EO; validation of IRT as a diagnostic discriminator between EO and testicular torsion requires a prospective comparative study design. Its incorporation as a complementary tool in the urgent evaluation of the pediatric acute scrotum is feasible, noninvasive, and clinically relevant.

## 1. Introduction

When a child presents to the emergency department with acute scrotal pain, the immediate and unavoidable priority is to exclude testicular torsion. However, in a considerable proportion of these cases the final diagnosis is epididymo‐orchitis (EO)—an inflammatory, and sometimes infectious, condition affecting the epididymis, the testis, or both structures simultaneously. Its reported annual incidence has been estimated at approximately 1.2 cases per 1000 boys 2–13 years of age [1], though the proportion of acute scrotum presentations ultimately diagnosed as EO varies widely across published series—from single digits to over half of cases—reflecting differences in population, age range, and diagnostic criteria across institutions [2]. EO and testicular torsion share clinical manifestations that are sufficiently similar to generate diagnostic uncertainty even in experienced hands [3].

The distinction between these two entities is not trivial. Torsion requires immediate surgical exploration; any delay translates into gonadal loss. EO, by contrast, responds to medical treatment, and operating unnecessarily on a child with EO carries an unjustified surgical risk. Given how frequently EO accounts for presentations of acute scrotal pain in children, distinguishing it reliably from torsion remains a daily diagnostic challenge at tertiary pediatric centers [3].

Scrotal Doppler ultrasonography (US) is currently the first‐line imaging study in the evaluation of the pediatric acute scrotum. It allows visualization of testicular blood flow with reasonable accuracy under optimal conditions. In real‐world emergency practice, however, its limitations are well recognized: the study is operator dependent, equipment may not be available around the clock, and—perhaps most importantly—in the first hours of symptom onset, the increase in vascularity typical of inflammation may not yet be sufficiently developed to yield a conclusive report [4]. This early‐presentation limitation has been repeatedly noted in the pediatric literature on sonographic differentiation of the acute scrotum [3].

Infrared thermography (IRT) operates on a straightforward pathophysiological principle: inflamed tissue generates more heat than healthy tissue and that temperature difference can be captured by cameras sensitive to infrared radiation [5]. The technique requires no contact, exposes the patient to no radiation, requires no sedation, and can be performed in minutes. In adult urology, IRT has been evaluated for varicocele and postoperative follow‐up [6]. In the pediatric scrotal setting, its exploration is more recent, with early prospective work suggesting feasibility as an adjunct in the evaluation of acute scrotal pain [7].

In our daily practice at CMN La Raza—a high‐volume tertiary referral center in Mexico City—we identified the need for a rapid, objective, and reproducible tool to support clinical assessment, particularly in cases where ultrasound fails to provide a definitive answer. It was from this practical need that we began to systematically incorporate IRT into the initial evaluation of these patients.

Published evidence on scrotal thermography in pediatrics remains scarce. Reference values for the thermal differential in pediatric EO are not well established, and no data exist characterizing how this differential evolves as a function of the number of days of clinical evolution. The present study does not aim to validate IRT as a diagnostic discriminator between EO and testicular torsion in undifferentiated acute scrotum; rather, it describes and characterizes the thermal pattern of confirmed pediatric EO—with the within‐patient thermal differential as the central outcome—and explores its evolution over time and its relationship to Doppler ultrasound findings. The present study reports results obtained at our institution between January 2024 and January 2026.

### 1.1. Objectives

To determine whether a statistically significant thermal differential exists between the EO‐affected testis and the contralateral healthy testis, to analyze whether this differential varies as a function of days of clinical evolution, and to explore the extent to which thermographic findings are concordant with or complementary to the results of scrotal Doppler US.

## 2. Material and Methods

### 2.1. Study Design

A prospective, descriptive, longitudinal, and comparative clinical study was conducted at the Pediatric Surgery and Pediatric Urology Services of Hospital General “Centro Médico Nacional La Raza” (CMN La Raza), IMSS, Mexico City, from January 1, 2024, to January 31, 2026. Each patient was systematically identified and enrolled at the time of presentation, with contemporaneous recording of all variables. The protocol was approved by the Local Research and Health Research Ethics Committee of CMN La Raza (institutional registration number R‐2024‐3502‐063) and was conducted in accordance with the principles of the Declaration of Helsinki. Written informed consent was obtained from the legal guardian, and assent was obtained from patients aged 12 years and older.

### 2.2. Participants

Inclusion criteria were as follows: male patients aged 1–17 years and 11 months with a clinical diagnosis of acute EO (acute or subacute scrotal pain with local inflammatory signs, with or without fever), established by the attending emergency physician, pediatric surgeon, or pediatric urologist; and informed consent from the guardian with age‐appropriate assent when applicable. Although the protocol permitted enrollment from 1 year of age, the youngest patient actually enrolled was 6 years old, reflecting the epidemiological reality that prepubertal EO is uncommon at this center during the study period.

Exclusion criteria were as follows: final diagnosis of testicular torsion, history of previous scrotal or inguinal surgery, active scrotal dermatopathy, thermographic images of insufficient technical quality, and refusal to participate by the guardian or patient.

### 2.3. Thermographic Acquisition Protocol

Each examination was performed in a room with an ambient temperature stabilized between 21°C and 23°C. Patients were asked to rest for a minimum of 10 min in the examination room prior to image acquisition to allow cutaneous thermal equilibration. The patient remained at rest in the supine position with the scrotal region exposed. The TLANESIS thermographic sensor (TLANESIS Medical Technologies, Mexico City, Mexico; model T‐1 Pro; CE‐marked for clinical use; COFEPRIS registration NOM‐241‐SSA1) was employed. This device has a thermal resolution of 0.1°C, a spectral range of 8–14 μm, and operates with a fixed skin emissivity value of 0.98, consistent with standard thermographic practice for human tissue. Images were obtained in the anterior projection of the scrotum, capturing both testes simultaneously at a distance of 30 cm from the scrotal surface. Raw thermographic data and image storage were managed within the device’s proprietary mobile application (TLANESIS App, v2.3); statistical analysis was performed separately using Python 3.11 with the pandas and SciPy libraries. No topical substance was applied prior to the study.

Two regions of interest (ROI) were manually delineated per testis: one at the upper pole and one at the lower pole. The reported value corresponds to the mean temperature of the delineated area. The assignment of the affected side was made exclusively by the attending clinician, without the thermography team having access to that information prior to image acquisition.

### 2.4. Scrotal Doppler US

Scrotal ultrasound was performed by specialist radiologists using a high‐frequency linear transducer (7.5–15 MHz) with color Doppler mode. Sonographic reports were categorized as positive for EO (epididymal thickening with altered—decreased, normal, or increased—testicular blood flow) or nondiagnostic (normal or nonspecific findings).

### 2.5. Variables

Primary outcome variables were as follows: thermographic temperature at the lower and upper poles of the healthy and affected testes, thermal differential (ΔT = temperature of affected testis − temperature of healthy testis) at each pole, number of complete days of evolution from symptom onset, and ultrasound result. Secondary variables were as follows: age in years and laterality of the affected testis.

### 2.6. Statistical Analysis

Data were processed using Python 3.11 (Pandas and SciPy libraries). Continuous variables were described as mean, standard deviation (SD), 95% confidence interval (CI), minimum, and maximum. The comparison of temperatures between the affected and healthy testes was performed using the paired Student’s *t*‐test since each patient served as his own control. Pearson’s correlation coefficient was calculated between the thermal differential and days of evolution. Agreement between IRT findings and scrotal Doppler ultrasound results was evaluated using Cohen’s kappa coefficient. Categorical variables were described using absolute frequencies and percentages. Statistical significance was set at *p* < 0.05.

## 3. Results

### 3.1. Demographic and Clinical Characteristics

During the study period, 72 patients meeting the inclusion criteria were enrolled. Mean age was 13.58 years (SD ± 2.56), with a range of 6–17 years and a median of 14 years. The 15‐year‐old age group was the most represented (18 patients, 25%), followed by the 14‐year‐old group (12 cases, 16.7%). Boys aged 6 and 8 years accounted for only 2 patients each. The left testis was most frequently affected (39 cases, 54.2%), followed by the right testis in 31 patients (43.1%), with bilateral involvement in only 2 cases (2.8%). The 2 bilateral cases were included in the study; for these patients, the thermographically warmer side was designated as the primary “affected” testis for comparative analysis, and both cases demonstrated positive thermal differentials consistent with the remainder of the sample. Demographic characteristics are summarized in Table 1.

**TABLE 1:** Demographic and clinical characteristics of the study sample (*n *= 72).

Characteristic	*n*	%	Statistic
Total patients	72	100%	—
Mean age (years)	13.58	—	SD ± 2.56
Age range	6–17 years	—	Median: 14 years
Left testis affected	39	54.2%	—
Right testis affected	31	43.1%	—
Bilateral involvement	2	2.8%	—
Positive ultrasound (EO)	61	84.7%	—
Nondiagnostic ultrasound	11	15.3%	—

*Note:* —: not applicable.

Abbreviations: EO, epididymo‐orchitis; SD, standard deviation.

### 3.2. Thermographic Temperature Analysis

The healthy testis showed a mean thermographic temperature of 32.28°C at both poles (SD ± 0.74°C; 95% CI: 32.11°C–32.45°C)—a value that was homogeneous across the entire sample. The affected testes recorded markedly higher temperatures: 33.96°C at the lower pole (SD ± 0.93°C; 95% CI: 33.74°C–34.18°C) and 34.42°C at the upper pole (SD ± 0.72°C; 95% CI: 34.25°C–34.59°C). The identical mean temperatures reported for the healthy testis at both poles reflect the high thermal homogeneity of the contralateral side across our sample. Differences were statistically significant at *p* < 0.001 for both poles (paired t‐test). Mean ΔT was 1.68°C at the lower pole (SD ± 1.20°C) and 2.14°C at the upper pole (SD ± 0.84°C), with individual values ranging from −0.10°C to 4.46°C at the lower pole and from 0.14°C to 4.33°C at the upper pole (Figure 1).

**FIGURE 1:**
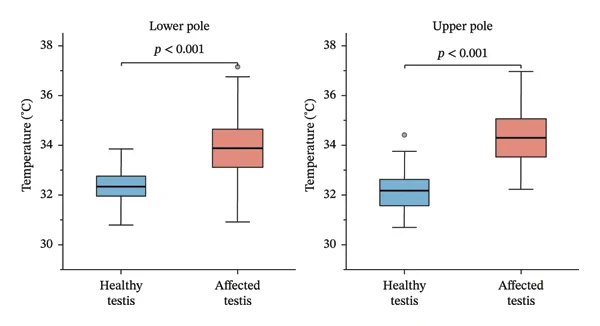
Box plots comparing thermographic temperatures of the healthy testis (blue) and the affected testis (red) at the lower pole (left panel) and upper pole (right panel). Horizontal bars with *p* < 0.001 denote statistical significance by paired Student’s *t*‐test. Circles represent outliers. *n* = 72.

### 3.3. Thermal Differential According to Days of Clinical Evolution

At 2 days from symptom onset, ΔT was 1.46°C at the lower pole and 1.78°C at the upper pole—values that already exceed the 1.0°C threshold proposed in the literature as the minimum clinically relevant cutoff. Differentials remained within similar ranges between Days 3 and 6, with the highest values recorded on Day 7: 3.04°C at the lower pole and 2.87°C at the upper pole. The Pearson correlation coefficient between days of evolution and ΔT was *r* = 0.24 (*p* = 0.04) for the lower pole and *r* = 0.25 (*p* = 0.03) for the upper pole, indicating a weak but statistically significant positive association (Figure 2 and Table 2).

**FIGURE 2:**
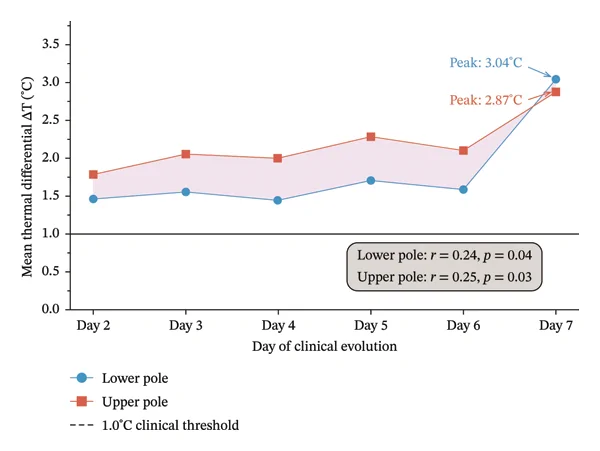
Mean thermal differential (ΔT = affected testis temperature − healthy testis temperature) at the lower pole (blue circles) and upper pole (red squares), stratified by day of clinical evolution. The dashed line marks the 1.0°C clinical threshold proposed in the literature. Pearson correlation coefficients: lower pole *r* = 0.24 (*p* = 0.04) and upper pole *r* = 0.25 (*p* = 0.03). *n* = 72.

**TABLE 2:** Mean thermal differential (ΔT) between affected and healthy testes by pole, stratified by day of clinical evolution.

Day of evolution	ΔT lower pole (°C)	ΔT upper pole (°C)	Comment
Day 2	1.46	1.78	Both poles exceed 1.0°C threshold
Day 3	1.55	2.05	—
Day 4	1.44	1.99	—
Day 5	1.71	2.28	—
Day 6	1.59	2.09	—
Day 7	3.04	2.87	Peak thermal response (both poles)

*Note:* ΔT: thermal differential (affected testis − healthy testis) in °C. Pearson *r* = 0.24–0.25, *p* < 0.05 for both poles.

### 3.4. Concordance With Scrotal Doppler US

Of the 72 patients, 61 (84.7%) had a positive sonographic report for EO, while in 11 cases (15.3%), the ultrasound was nondiagnostic. All 11 of these patients had been assigned a clinical diagnosis of acute EO by the attending physician prior to enrollment. Confirmation of EO in this subgroup was supported by clinical resolution with antibiotic and anti‐inflammatory therapy in 10 patients (90.9%), documented within 7–14 days of follow‐up, and repeat scrotal ultrasound at 7 days demonstrating epididymal thickening consistent with resolving EO in the remaining patient (9.1%). No patient in this subgroup underwent surgical exploration or received an alternative diagnosis during follow‐up. In these 11 patients, thermography documented a mean temperature of 34.22°C (SD ± 1.34°C; 95% CI: 33.32°C–35.12°C) at the lower pole of the affected testis, with a mean ΔT of 1.92°C relative to the contralateral testis. In every single one of these cases, without exception, the temperature of the affected testis exceeded that of the healthy testis, even when ultrasound had been unable to confirm it.

## 4. Discussion

The data from this series directly address the central question of the study: a statistically significant temperature difference exists between the EO‐affected testis and the contralateral healthy testis, and this difference is detectable by IRT. A mean differential of 1.68°C at the lower pole and 2.14°C at the upper pole, with *p* < 0.001 at both sites, can scarcely be attributed to chance. It is important to emphasize that this study is best interpreted as a descriptive characterization of the thermal pattern of pediatric EO rather than a validation of IRT as a diagnostic discriminator between EO and testicular torsion in undifferentiated acute scrotum. Torsion cases were not enrolled as a comparator group; all patients were already clinically diagnosed with EO prior to enrollment. The within‐patient thermal differential is the central and most robust finding of this study (Tables 3 and 4).

**TABLE 3:** Thermographic temperature comparisons between the affected and healthy testes by pole (*n* = 72).

Measurement	Affected testis mean (°C)	Healthy testis mean (°C)	Mean ΔT (°C)	*p* value
Lower pole temperature	33.96 ± 0.93	32.28 ± 0.74	1.68 ± 1.20	< 0.001
Upper pole temperature	34.42 ± 0.72	32.28 ± 0.74	2.14 ± 0.84	< 0.001

*Note:* Values expressed as mean ± standard deviation. ΔT: thermal differential (affected testis − healthy testis). *p* values from paired Student’s *t*‐test.

**TABLE 4:** Thermographic comparison between patients with positive and nondiagnostic scrotal Doppler ultrasound findings.

Ultrasound result	*n* (%)	Mean affected testis temp.: lower pole (°C)	Mean ΔT (°C)
Epididymo‐orchitis (positive)	61 (84.7%)	33.91	1.63
Nondiagnostic	11 (15.3%)	34.22 ± 1.34	1.92

*Note:* ΔT: mean thermal differential (affected testis − healthy testis). In all 11 nondiagnostic ultrasound cases, the affected testis temperature exceeded the healthy testis temperature.

Comparable pediatric thermographic threshold data are not yet well established in the published literature, which limits direct comparison of our ΔT findings against externally validated cutoffs. The mean thermal differentials observed in our cohort, 1.68°C at the lower pole and 2.14°C at the upper pole, represent, to our knowledge, one of the more systematic descriptions of this parameter in a pediatric EO population to date, underscoring the need for prospective multicenter data to establish validated reference thresholds.

The behavior of the thermal differential as a function of days of evolution is clinically meaningful. The Pearson correlation obtained (*r* ≈ 0.24–0.25) is weak—meaning that days of evolution alone do not reliably predict the magnitude of the differential—but the marked increase at Day 7 (ΔT > 3°C at both poles) suggests that the inflammatory response reaches its peak thermal expression within that time window. The number of patients contributing data at each day interval was as follows: Day 2 (*n* = 14), Day 3 (*n* = 18), Day 4 (*n* = 16), Day 5 (*n* = 9), Day 6 (*n* = 7), and Day 7 (*n* = 8). Although the Day‐7 subgroup is relatively small and these data should be interpreted with caution, the peak ΔT is biologically plausible. Acute EO is characterized by a cascade of vasodilatation, increased vascular permeability, and local cytokine release; this process does not necessarily resolve as quickly as clinical symptoms improve, and it is biologically plausible that tissue hyperemia could remain elevated for several days after symptom onset before an anti‐inflammatory phase begins to predominate. Even when clinical symptoms may subjectively ease by Day 7, the underlying inflammatory vascular response may still be near its maximum thermal expression. We offer this as a plausible physiological explanation for our observation rather than a validated finding since EO‐specific data on the time course of thermal resolution are not yet available in the pediatric literature.

The subgroup with nondiagnostic ultrasound merits special attention, representing 15.3% of the sample, consistent with the well‐recognized limitation that a meaningful proportion of pediatric scrotal Doppler studies remain equivocal, particularly when patients are evaluated in the first hours of presentation [3]. All 11 patients in this subgroup had a clinical diagnosis of EO prior to enrollment, and EO was subsequently confirmed in all cases through clinical resolution with antibiotic and anti‐inflammatory therapy (10 patients) or repeat sonographic evidence at Day 7 (1 patient). In these patients, thermography demonstrated a positive thermal differential in 100% of the cases, suggesting that the thermal tissue response precedes detectable Doppler changes. It is important to note that the present study design does not allow us to claim that thermography “detected what ultrasound missed” in a formal diagnostic accuracy sense since torsion cases were not included as a comparator arm. Rather, the finding demonstrates that IRT is capable of documenting objective thermal asymmetry consistent with inflammation even when Doppler ultrasound is nondiagnostic, making it a clinically useful complementary tool. This does not mean that IRT should replace ultrasound but rather that it may be most useful precisely when ultrasound fails.

From a practical standpoint, once the acquisition protocol is standardized, thermography is considerably less dependent on individual technical skill than Doppler US, a relevant consideration in a high‐volume hospital such as CMN La Raza. Emerging prospective work has begun to explore infrared thermal imaging as a rapid adjunct at the point of triage for acute scrotal pain [7]. Our data, in which virtually, the entire sample showed a positive thermal differential, support the feasibility of incorporating a similar triage‐oriented approach at our tertiary care setting although prospective evaluation of its impact on time‐to‐diagnosis is still needed.

This study has several limitations. It is a single‐center observational study, which restricts the generalizability of findings. The study includes only patients already clinically diagnosed with EO; testicular torsion cases were not enrolled as a comparator group. Consequently, the study characterizes the thermal pattern of EO but does not validate IRT as a diagnostic discriminator between EO and testicular torsion in undifferentiated acute scrotum. The design did not permit formal calculation of sensitivity and specificity for IRT, as a uniform reference standard was not available for all cases. Microbiological confirmation was unavailable in the majority of patients, precluding distinction between bacterial, viral, and reactive etiologies. The ambient temperature range of 21°C–23°C is slightly broader than some standard thermography protocols recommend; however, because the study employs simultaneous bilateral imaging with each patient acting as his own control, intersession ambient variability has a diminished effect on the within‐patient thermal differential. ROI delineation was performed manually by the thermography team, and interobserver reproducibility of this process was not formally assessed; this represents an additional source of potential measurement variability that should be addressed in future studies through standardized or semiautomated ROI segmentation protocols. It should also be acknowledged that elevated scrotal temperature is not specific to EO. False‐positive findings could theoretically arise in other conditions that increase scrotal temperature, including postdetorsion hyperemia, idiopathic scrotal edema, cellulitis, scrotal involvement in Henoch–Schönlein purpura, or other inflammatory processes. These potential confounders reinforce the importance of using IRT as a complementary rather than standalone diagnostic tool, always in conjunction with clinical evaluation and, where possible, Doppler US.

## 5. Conclusions

IRT detected a statistically significant temperature increase in the EO‐affected testes relative to the contralateral healthy testes. The mean thermal differentials documented—1.68°C at the lower pole and 2.14°C at the upper pole—exceeded the 1.0°C threshold identified as clinically relevant in the literature. This study characterizes the thermal pattern of pediatric EO; the within‐patient thermal differential is a reproducible and objective finding that supports the applicability of this technique as a complementary tool in the primary evaluation of pediatric patients presenting to the emergency department.

Incorporating IRT as part of the initial assessment protocol for the pediatric acute scrotum at CMN La Raza is feasible, safe, and clinically justified on the basis of these results. The next logical step is the design of a multicenter prospective study to calculate the sensitivity, specificity, and predictive values of a defined ΔT cutoff with greater precision.

## Author Contributions

José Alfredo León Zamudio: conceptualization, study design, data collection, data curation, formal analysis, original manuscript drafting, review and editing, project administration, and supervision. Onésimo Avilés González: conceptualization, patient recruitment, clinical evaluation, data collection, and review and editing. Manuel López: critical review of important intellectual content, expert consultation in Pediatric Urology, and review and editing. Alma Patricia González: data collection, methodological design, statistical analysis, and review and editing.

## Funding

This research received no external funding.

## Ethics Statement

The study was approved by the Local Research and Health Research Ethics Committee of CMN La Raza, IMSS (registration number R‐2024‐3502‐063). All procedures were conducted in accordance with the Declaration of Helsinki. Written informed consent was obtained from legal guardians, and age‐appropriate assent was obtained from patients aged ≥ 12 years.

## Conflicts of Interest

The authors declare no conflicts of interest.

## Supporting Information

Additional supporting information can be found online in the Supporting Information section.

## Supporting information


**Supporting Information** STROBE Checklist Advances Urology.

## Data Availability

The data that support the findings of this study are available from the corresponding author upon reasonable request, subject to institutional data‐use policies.
