# Supplementary material for: Infrared Thermography as a Complementary Diagnostic Tool in Pediatric Acute Epididymo‐Orchitis: Characterization of the Thermal Differential Pattern
Source: Adv Urol. 2026 Nov 13;2026:5411884. doi: 10.1155/aiu/5411884 (PMC13768804; doi:10.1155/aiu/5411884)
Supplement: Supplementary file 1 [file AIU-2026-5411884-s001.docx]

**STROBE Checklist — Strengthening the Reporting of Observational Studies in Epidemiology**

**Study type: Prospective cohort / observational study**

**Manuscript title:** *Infrared Thermography as a Complementary Diagnostic Tool in Pediatric Acute Epididymo-orchitis: Differential Thermal Analysis*

**Authors:** León Zamudio JA, Avilés González O, López M, González AP

**Journal:** *Advances in Urology*

**Date:** April 15, 2026

**Corresponding author:** Dr. José Alfredo León Zamudio — Josealfredoleonzam@gmail.com

*Note: This checklist has been completed in accordance with the STROBE Statement for observational studies (von Elm et al., 2007; Lancet 2007;370:1453–57). All 22 items are addressed in the submitted manuscript. Item locations refer to the main manuscript sections.*

| **Item** | **Recommendation (STROBE — Cohort/Cross-sectional)** | **Section / Page** | **Reported** |
| --- | --- | --- | --- |
| **No.** |  | **in manuscript** | **Yes / No / NA** |
| **TITLE AND ABSTRACT** | | | |
| 1 | Indicate the study's design with a commonly used term in the title or the abstract. Indicate the study's design in the title or abstract. | Title page & Abstract (Methods paragraph) | ✓ |
| **INTRODUCTION** | | | |
| 2 | Background/rationale: Explain the scientific background and rationale for the investigation being reported. | Section 1 — Introduction, paragraphs 1–5 | ✓ |
| 3 | Objectives: State specific objectives, including any prespecified hypotheses. | Section 1.1 — Objectives | ✓ |
| **METHODS** | | | |
| 4 | Study design: Present key elements of study design early in the paper. | Section 2.1 — Study design | ✓ |
| 5 | Setting: Describe the setting, locations, and relevant dates, including periods of recruitment, exposure, follow-up, and data collection. | Section 2.1 — Study design (Jan 2024 – Jan 2026; CMN La Raza, IMSS) | ✓ |
| 6 | Participants: Give the eligibility criteria, and the sources and methods of selection of participants. | Section 2.2 — Participants (inclusion & exclusion criteria) | ✓ |
| 7 | Variables: Clearly define all outcomes, exposures, predictors, potential confounders, and effect modifiers. Give diagnostic criteria, if applicable. | Section 2.5 — Variables | ✓ |
| 8 | Data sources/measurement: For each variable of interest, give sources of data and details of methods of assessment (measurement). Describe comparability of assessment methods if there is more than one group. | Sections 2.3 (thermography protocol) & 2.4 (ultrasound) | ✓ |
| 9 | Bias: Describe any efforts to address potential sources of bias. | Section 2.3 (blinded side assignment); Section 6 — Limitations | ✓ |
| 10 | Study size: Explain how the study size was arrived at. | Section 2.1 (consecutive enrollment, 72 patients over 2 years) | ✓ |
| 11 | Quantitative variables: Explain how quantitative variables were handled in the analyses. If applicable, describe which groupings were chosen and why. | Section 2.6 — Statistical analysis | ✓ |
| 12 | Statistical methods: (a) Describe all statistical methods, including those used to control for confounding. (b) Describe any methods used to examine subgroups and interactions. (c) Explain how missing data were addressed. (d) If applicable, describe analytical methods taking account of sampling strategy. (e) Describe any sensitivity analyses. | Section 2.6 — Statistical analysis (paired t-test, Pearson r, Python 3.11) | ✓ |
| **RESULTS** | | | |
| 13 | Participants: Report the numbers of individuals at each stage of the study (e.g., numbers potentially eligible, examined for eligibility, confirmed eligible, included in the study, completing follow-up, and analysed). Give reasons for non-participation at each stage. Consider use of a flow diagram. | Section 3.1 — Demographic and clinical characteristics; Table 1 | ✓ |
| 14 | Descriptive data: Give characteristics of study participants (e.g., age, sex) and information on exposures and potential confounders. | Section 3.1 — Demographic and clinical characteristics; Table 1 | ✓ |
| 15 | Outcome data: Report numbers of outcome events or summary measures. | Section 3.2 — Thermographic temperature analysis; Table 2; Figure 1 | ✓ |
| 16 | Main results: (a) Give unadjusted estimates and, if applicable, confounder-adjusted estimates and their precision (e.g., 95% CI). (b) Report category boundaries when continuous variables were categorized. (c) If relevant, consider translating estimates of relative risk into absolute risk for a meaningful time period. | Sections 3.2, 3.3, 3.4; Tables 2–4; Figures 1–2 | ✓ |
| 17 | Other analyses: Report other analyses done — e.g., analyses of subgroups and interactions, and sensitivity analyses. | Section 3.4 — Non-diagnostic ultrasound subgroup; Table 4 | ✓ |
| **DISCUSSION** | | | |
| 18 | Key results: Summarise key results with reference to study objectives. | Section 4 — Discussion, paragraph 1 | ✓ |
| 19 | Limitations: Discuss limitations of the study, taking into account sources of potential bias or imprecision. | Section 4 — Discussion, final paragraph; Section 6 — Limitations | ✓ |
| 20 | Interpretation: Give a cautious overall interpretation of results considering objectives, limitations, multiplicity of analyses, results from similar studies, and other relevant evidence. | Section 4 — Discussion, paragraphs 2–4 | ✓ |
| 21 | Generalisability: Discuss the generalisability (external validity) of the study results. | Section 6 — Limitations (single-center design noted) | ✓ |
| **OTHER INFORMATION** | | | |
| 22 | Funding: Give the source of funding and the role of the funders for the present study and, if applicable, for the original study on which the present article is based. | Funding section (no external funding) | ✓ |

**Reference:** von Elm E, Altman DG, Egger M, Pocock SJ, Gøtzsche PC, Vandenbroucke JP; STROBE Initiative. The Strengthening the Reporting of Observational Studies in Epidemiology (STROBE) statement: guidelines for reporting observational studies. *Lancet.* 2007;370(9596):1453–57. https://doi.org/10.1016/S0140-6736(07)61602-X
